# The Role of Macrophage Migration Inhibitory Factor (MIF) and D-Dopachrome Tautomerase (D-DT/MIF-2) in Infections: A Clinical Perspective

**DOI:** 10.3390/biomedicines12010002

**Published:** 2023-12-19

**Authors:** David Breidung, Ioannis-Fivos Megas, David Lysander Freytag, Jürgen Bernhagen, Gerrit Grieb

**Affiliations:** 1 Department of Plastic, Reconstructive and Hand Surgery, Burn Center for Severe Burn Injuries, Klinikum Nuremberg Hospital, Paracelsus Medical University, Breslauer Str. 201, 90471 Nuremberg, Germany; david.breidung@icloud.com; 2 Department of Orthopaedic and Trauma Surgery, Center of Plastic Surgery, Hand Surgery and Microsurgery, Evangelisches Waldkrankenhaus Spandau, Stadtrandstr. 555, 13589 Berlin, Germany; fivos.megas@gmail.com; 3 Department of Plastic Surgery and Hand Surgery, Gemeinschaftskrankenhaus Havelhoehe, Kladower Damm 221, 14089 Berlin, Germany; davidlysander.freytag@havelhoehe.de; 4 Division of Vascular Biology, Institute for Stroke and Dementia Research (ISD), Klinikum der Universität München (KUM), Ludwig-Maximilians-University (LMU), Feodor-Lynenstraße 17, 81377 Munich, Germany; juergen.bernhagen@med.uni-muenchen.de; 5 Munich Cluster for Systems Neurology (SyNergy), Feodor-Lynenstraße 17, 81377 Munich, Germany; 6 Department of Plastic Surgery and Hand Surgery, Burn Center, Medical Faculty, RWTH Aachen University, Pauwelsstrasse 30, 52074 Aachen, Germany

**Keywords:** macrophage migration inhibitory factor, MIF, infectious disease, sepsis, biomarker, cytokine

## Abstract

Macrophage migration inhibitory factor (MIF) and its homolog, D-dopachrome tautomerase (D-DT), are cytokines that play critical roles in the immune response to various infectious diseases. This review provides an overview of the complex involvement of MIF and D-DT in bacterial, viral, fungal, and parasitic infections. The role of MIF in different types of infections is controversial, as it has either a protective function or a host damage-enhancing function depending on the pathogen. Depending on the specific role of MIF, different therapeutic options for MIF-targeting drugs arise. Human MIF-neutralizing antibodies, anti-parasite MIF antibodies, small molecule MIF inhibitors or MIF-blocking peptides, as well as the administration of exogenous MIF or MIF activity-augmenting small molecules have potential therapeutic applications and need to be further explored in the future. In addition, MIF has been shown to be a potential biomarker and therapeutic target in sepsis. Further research is needed to unravel the complexity of MIF and D-DT in infectious diseases and to develop personalized therapeutic approaches targeting these cytokines. Overall, a comprehensive understanding of the role of MIF and D-DT in infections could lead to new strategies for the diagnosis, treatment, and management of infectious diseases.

## 1. Introduction

In recent years, the field of infectious diseases has become increasingly important due to the emergence of new pathogens, the re-emergence of older iterations, as well as the development of antibiotic resistance [1,2,3]. The human immune system plays an important role in combating these invading pathogens by recognizing and destroying them before they have an opportunity to cause harm [4]. Understanding the intricate workings of the individual components of the human immune system, which encompass a wide range of defenses such as cytokines, chemokines, antibodies, and other small molecules, is critical to establishing a solid basis for the prevention and treatment of infectious diseases. Sepsis is a life-threatening complication of an infection that affects millions of people worldwide annually [5,6]. Despite advances in supportive care, sepsis remains a major cause of morbidity and mortality [5,6,7].

Besides the adaptive immune system, the innate, or nonspecific, immune system represents one of the two primary immunity strategies in humans. This immune system has evolved through small refinements over generations to its present form and is encoded in our germline [8,9]. It represents the first-line protection against exogenous microorganisms [10]. Phagocytic cells, the complement system, and Toll-like receptors comprise the innate immune system, among a variety of other factors [8].

**Figure 1:**
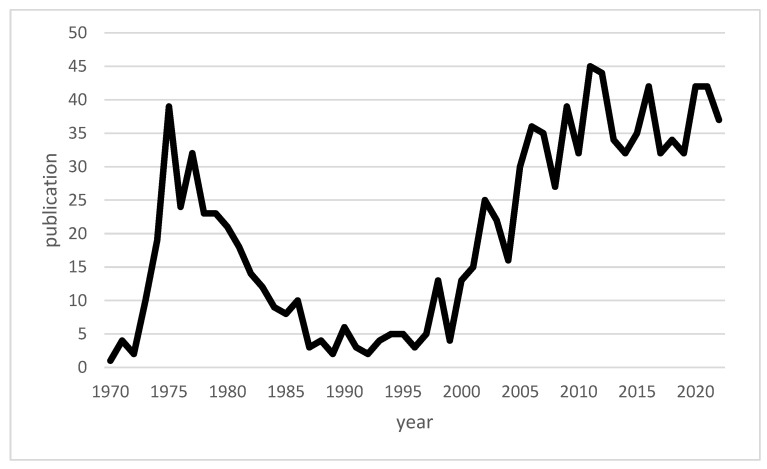
Line chart of the publications on “macrophage inhibitory factor” and “infections” available across time.

**Figure 2:**
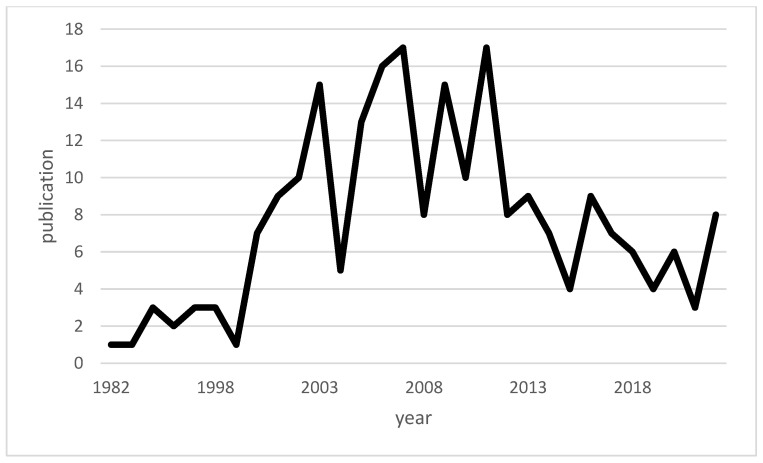
Line chart of the publications on “macrophage inhibitory factor” and “sepsis” available across time.

Macrophage migration inhibitory factor (MIF) is a pleiotropic cytokine produced by a variety of cells in the body, including immune cells such as macrophages. The discovery of MIF dates back to the results of two independent studies in 1966. At that time, John David and Barry Bloom described a factor produced by T cells and associated with the inhibition of macrophage migration in delayed-type hypersensitivity [11,12,13]. Since the discovery of MIF, a number of studies on MIF have been published with varied focuses, and MIF has been transformed within scientific discourse from a mysterious cytokine into a recognized, important component of the innate immune system [14]. Figure 1 shows a timeline of available publications on PubMed for “macrophage inhibitory factor” and “infections”. Figure 2 illustrates the number of publications per year for “macrophage inhibitory factor” and “sepsis” on PubMed. An increase in the number of publications on MIF in infections has occurred since approximately the turn of the millennium, whereas publications on sepsis peaked in the first decade of the 21st century. While initial focus on MIF in sepsis yielded valuable insights [15,16], subsequent research may have diversified to explore MIF’s involvement in a broader spectrum of infections, reflecting the dynamic nature of scientific inquiry and the recognition of MIF’s significance beyond sepsis.

MIF is a cytokine that plays a key role in the regulation of immune responses and inflammation [14]. Besides its role in delayed-type hypersensitivity, MIF also has a direct chemokine-like function and promotes cell migration [17]. This includes the dominant role of MIF in inflammatory and atherogenic leukocyte recruitment, a process that occurs based on an interaction between the CXC chemokine receptors CXCR2/CXCR4 and MIF as a non-cognate ligand [18]. CD74, CXCR2, CXCR4, and CXCR7 represent the membrane receptors to which MIF can bind [17]. Of these, CD74 is the most widely studied membrane receptor [17]. Through its binding to the extracellular domain of CD74, MIF triggers the activation of the extracellular signal-regulated kinase (ERK)-1/2 pathway, with CD44 serving as an essential component of the CD74 receptor complex that facilitates MIF signal transduction [19,20,21]. When MIF binds to CXCR2, it induces chemotactic responses, promoting the recruitment and migration of immune cells, including monocytes and neutrophils, to inflammatory sites [18]. This may contribute to the early immune response to infections. MIF also induces the targeted migration of T cells via CXCR4 [18], and is thus involved in the adaptive immune response to infections. The MIF/CXCR7 interaction has also been shown to be involved in inflammatory processes, particularly via B cell chemotaxis, as shown by Alampour-Rajabi et al. [22]. MIF also shows tautomerase activity using the substrates D-dopachrome, 4-hydroxyphenyl pyruvate, and phenyl pyruvate [23]. MIF’s pro-inflammatory role is linked to its tautomerase and oxidoreductase activities [24]. The tautomerase activity of MIF is notably involved in high-fat diet induced obesity, impacting inflammation [24]. Notably, the absence of tautomerase activity in MIF correlates with improved insulin resistance [24]. The tautomerase activity of MIF also plays a special role in the consideration of MIF in relation to infectious diseases. For example, a specific inhibitor of tautomerase activity could be identified as an antagonizing agent against MIF-mediated inflammatory processes by a reduction of inflammatory cytokines, cytotoxicity, cell death, and production of reactive oxygen species and thereby represent a possible supportive treatment in leptospirosis [25].

Research has also demonstrated that macrophage migration inhibitor factor (MIF), which is also referred to as parthanatos-associated apoptosis-inducing factor (AIF) nuclease, contributes as a key mediator of parthanatic cell death in acute disorders. Additionally, it plays a role in chronic neurodegenerative diseases such as Parkinson’s disease [26,27,28]. This effect is attributed to the nuclease activity of MIF [26,29].

In the human genome, the MIF gene is located on human chromosome 22q11.2. Two polymorphisms of this gene have been found to have associations with different diseases in humans [17,30,31,32,33]. From an evolutionary perspective, the MIF protein represents an ancient molecule with homologs in different prokaryotes, such as bacterial cells, as well as in eukaryotes, such as amphibians, helminths, and nematodes [34]. The MIF protein has a molecular mass of 12.5 kDa and consists of 114 amino acids [35,36]. The MIF cytokine family exerts a special role in infectious diseases and inflammations (see Table 1).

Aside from its role in infectious diseases, MIF plays a role in inflammatory diseases such as asthma or atherosclerosis, various forms of cancer, several autoimmune diseases, burn injuries, wound healing, and even free-flap ischemia [43,44,45,46,47,48,49,50,51,52,53,54,55,56,57]. The diverse functions of the MIF protein in the immune response of the organism to microbial pathogens have been demonstrated in various studies [37,39,41,58,59]. Whether the MIF protein acts as a protective factor or potentiator of a damaging host response depends on the specific pathogenic microorganism involved [41].

Numerous hypotheses have been proposed to explain the complex and dual role of MIF in different infectious diseases. In scenarios where MIF plays a protective role, MIF is thought to be a critical component of the early immune response [60]. MIF may enhance the activation of immune cells, particularly macrophages, and facilitate the release of pro-inflammatory cytokines, contributing to the efficient clearance of invading pathogens [61,62]. Conversely, the deleterious effects of MIF in other infections may be due to its potential to exacerbate inflammation and contribute to tissue damage. In chronic inflammation and autoimmune diseases, MIF is thought to play a role in maintaining inflammation [63], leading to persistent tissue damage and dysfunction. In addition, in certain viral infections such as HIV, MIF can create a microenvironment conducive to viral infection [62], allowing the virus to evade host defenses and cause persistent infections. The specific mechanisms behind these deleterious effects may be related to the dysregulation of immune responses, including the modulation of cytokine profiles and the promotion of immune cell susceptibility to viral invasion [64,65]. It should therefore be noted that the effect of MIF is context-dependent and varies depending on the pathogen, stage of infection, and general immunological milieu. One factor that could also influence in which infections MIF exerts a protective or deleterious effect is the affected organ, as MIF could also exert organ-specific protective effects in diseases [66].

Recently, another protein called D-dopachrome tautomerase (D-DT), which is structurally similar to MIF and also called MIF-2, has gained interest. D-DT also plays a role in the human immune response, but its precise functions are still being investigated. This review aims to provide an overview of MIF’s role in different types of infections to illustrate the interaction pathways and potential clinical applications of the macrophage migration inhibitory factor and the D-dopachrome tautomerase.

## 2. Bacterial Infections

MIF plays a complex role in the host response to bacterial infections. In their 2005 study, Oddo et al. outlined the role of MIF in response to *Mycobacterium tuberculosis* [67]. An increase in the release of MIF from macrophages in response to *Mycobacterium tuberculosis* was observed. The neutralization of MIF with anti-MIF antibodies in macrophages resulted in an enhancement in the growth of *Mycobacterium tuberculosis*. Additional administration of exogenous MIF led to an inhibition of mycobacterial growth. In conclusion, this study demonstrated a protective, bacterial growth inhibitory role of MIF in the immune response against *Mycobacterium tuberculosis*.

Das et al. showed in 2013 that the −794 CATT_5/5_ variant, which represents a low-expressor MIF genotype, is more frequently found in patients with a disseminated *Mycobacterium tuberculosis* infection than in disease-free control patients [41]. MIF-deficient mice also exhibit increased pulmonary pathology and earlier mortality in response to *Mycobacterium tuberculosis* infection compared to wild-type (WT) mice [41]. Shang et al. further demonstrated a potential use of serum MIF levels as a marker for monitoring treatment efficacy in active pulmonary tuberculosis [68].

In a study by Sumaiya et al. investigating MIF as a diagnostic marker for leptospirosis, elevated serum MIF levels were shown in cases of leptospirosis compared to uninfected control patients [69]. A receiver-operating characteristic curve analysis accordingly showed an area under the curve of >0.9 for differentiation between the two groups. In addition, the study reported a correlation of MIF levels with disease progression and severity.

Zhang et al. investigated the role of MIF in periodontitis in their study [70]. Assessing human immortalized oral epithelial cells, this study found that the secretion of MIF in the supernatant of *Porphyromonas gingivalis*-infected cells increased compared with that in uninfected control cells. Furthermore, MIF has been shown to be increased in both serum and gingival tissue of patients with periodontitis [70].

In their studies, Savva et al. and Kloek et al. showed the prognostic value of the identification of the genotype of MIF and the determination of MIF plasma levels, whereby high-expression MIF alleles as well as higher-plasma MIF concentrations were associated with a worsened clinical outcome in pneumococcal meningitis [71,72]. Neutralizing antibodies of MIF also improved bacterial clearance and reduced lethality in pneumococcal sepsis in mice [71]. A similar trend was observed in a study by Jose et al. on the role of MIF in *Clostridium difficile* infections [73]. Here, an improved outcome was seen in mice after the administration of anti-MIF antibodies compared to the use of control antibodies. The severity of symptoms was alleviated (shorter duration and less severe diarrhea) and mortality was lower after administration of anti-MIF antibodies.

These observations contrast with the role of MIF established in a study of *Salmonella typhimurium* infections [59]. Here, all MIF-deficient mice died within 25 days of per os infection. By comparison, 50% of control mice survived beyond 75 days. There was also increased bacterial growth in the visceral organs of the MIF-deficient mice. The levels of IFN-γ, IL-12, as well as TNF-α, were decreased in the post-infection phase in MIF-deficient mice compared with control mice. Thus, MIF can be considered a protective mediator of the immune response to *Salmonella typhimurium*.

Adamali et al. investigated the effect of tautomerase null gene MIF knock-in mice in *Pseudomonas aeruginosa* infections [74]. They found that tautomerase null mice had lower TNF-α and neutrophil levels and a lower bacterial load after infection.

Doroudian et al. investigated the effect of drug-loaded aerosol nanoparticles containing an MIF inhibitor on the inflammatory response against *Pseudomonas aeruginosa* [75]. The authors used SCD-19, a small molecule inhibitor of the tautomerase enzymatic activity of MIF, to limit MIF activity. MIF-deficient macrophages were found to exhibit enhanced killing of *Pseudomonas aeruginosa* compared with wild-type bone marrow-derived macrophages. The treatment of bone marrow-derived macrophages with SCD-19 resulted in enhanced bactericidal activity against *Pseudomonas aeruginosa*. Furthermore, adding recombinant MIF to human airway epithelial cells promoted biofilm formation in a laboratory strain of *Pseudomonas aeruginosa*, while SCD-19 inhibited attachment to epithelial cells. Thus, the study by Doroudian et al. demonstrates that aerosolized drug delivery systems targeting MIF are a potential treatment option for *Pseudomonas aeruginosa* infections and cystic fibrosis lung disease.

Consequently, the tautomerase activity of MIF and its ability to promote bacterial biofilm formation appear to be key aspects of Pseudomonas infections [76]. Overall, the role of MIF in bacterial infections is complex and depends on the type of bacteria and the stage of infection. Although they act as a protective factor in a variety pathogens, elevated MIF levels tend to worsen prognosis in pneumococcal pneumonia, for example. MIF variants have also been implicated in the pathogenesis of different bacteria. Other possible uses have been demonstrated beyond their potential therapeutic applications, such as in therapy monitoring and the prediction of clinical course. An overview of the partially opposing effects of MIF in the various bacterial infections is shown in Table 2. The table demonstrates that whether MIF has a protective or deleterious effect is not solely determined by the Gram staining groups.

## 3. Viral Infections

In addition to its role in the interactions in bacterial infections, MIF plays a key role in the immune system’s response to viral infections. MIF is known to play a role in several stages of viral pathogenesis, with different viral pathogens showing varying profiles of MIF.

In dengue virus infections, serum MIF levels have been shown to correlate with disease severity, with higher levels in patients who died of dengue hemorrhagic fever (DHF) than in patients who survived DHF and patients with mild dengue fever [77]. MIF-deficient mice presented with lower viremia, reduced proinflammatory cytokine levels, and delayed lethality compared with WT mice [1]. A 2018 study by Chen et al. investigated the role of macrophage migration inhibitory factor (MIF) in nonstructural protein 1 (NS1)-induced glycocalyx degradation during dengue virus infection [78]. The results of this study suggest that MIF is directly involved in NS1-induced glycocalyx degradation, and that targeting MIF may be a potential therapeutic strategy for the prevention of vascular leakage induced by the dengue virus. In a 2020 review, Lai et al. outlined the three major pathogenic roles of MIF in dengue virus infection [79]: the facilitation of virus replication, a contribution to vascular leakage, and the modulation of immune cell function.

In a 2018 study, Trifone et al. presented the effect of MIF in monocyte-derived macrophages (MDMs) infected with human immunodeficiency virus type I (HIV) [62]. The stimulation of HIV-infected MDMs with MIF increased the induction of IL-1β, IL-6, IL-8, TNF-α, and sICAM. This effect was reversed with the application of a CD74-blocking antibody, except for in the case of sICAM. In addition to the increased production of cytokines, the predisposition of unactivated CD4^+^ T cells to HIV infection was shown. Thus, the interaction between MIF and CD74 could play a role in viral dissemination and reservoir seeding of HIV [62]. In 2022, Trifone et al. further demonstrated that MIF stimulation in HIV infection leads to an increased Th17-like cell profile and thus a permissive cell type toward HIV-1 [65].

In a study of verruca vulgaris, MIF levels of biopsies were compared between patients with common warts caused by human papillomavirus and a healthy control group [80]. One lesional and one perilesional biopsy were taken in the case group, and these were in turn extracted from the same sites in the control group. The MIF levels of the biopsies were increased in the case group compared with the control group. However, no significant difference was found between lesional and perilesional biopsies. Therefore, it can be asserted that MIF plays a role in the development of HPV-infected cells into common warts [80].

Smith et al. showed in their study that MIF-deficient mice have a reduced viral load, less inflammation, and lower mortality from influenza A virus (IAV) compared to WT mice [81]. Treatment with an anti-MIF antibody improved survival after IAV infection. In comparison, de Souza et al. demonstrated in a study that MIF plays an important role in viral clearance in respiratory syncytial virus (RSV) infections and that the inhibition of MIF in mice macrophages is associated with an increase in viral load [82]. Interestingly, according to the current state of the literature, this effect appears to be unique in the field of viral infections. Furthermore, in this study, it could be demonstrated that the induction of MIF expression by RSV is dependent on the generation of reactive oxygen species (ROS) through the activation of NADPH oxidase, similar to the mechanism observed for *T. gondii*-triggered MIF expression [82,83].

Recently, Dheir et al. presented a study evaluating the prognostic significance of MIF in pneumonia caused by severe acute respiratory syndrome coronavirus 2 (SARS-CoV-2) [84]. On average, MIF levels were found to be elevated in patients in an intensive care unit (ICU) compared with patients in a normal unit. However, the discriminatory power of MIF between ICU and normal ward patients in this study was lower than that of other blood biomarkers, such as D-Dimer, Troponin or Ferritin. Aksakal et al. also reported the diagnostic and prognostic value of MIF in SARS-CoV-2 infections [85]. Serum MIF levels were higher in infected patients compared with the control group, and serum MIF levels were higher in severely than moderately ill patients in the study.

Table 3 provides an overview of the role of MIF in different viral infections. These findings collectively demonstrate that MIF can enhance viral replication, promote viral persistence by inhibiting host immune responses, or improve viral clearance by modulating host immune responses. Hypothesizing the dualistic role of MIF in viral infections, we suggest that the specific effect of MIF may depend on the interplay between MIF and the unique pathogenesis of the virus in question. In certain viral infections, MIF could have a protective effect by enhancing early immune responses and thus supporting virus clearance. Conversely, in other infections, MIF could contribute to deleterious effects, potentially promoting chronic inflammation and tissue damage. This indicates that the nuanced effects of MIF in viral diseases may be shaped by the complex dynamics of host–virus interaction, immune modulation, and the specific organ tropism of the infectious agent. Based on this, it could be concluded that MIF-targeting drugs have potential therapeutic applications in the treatment of some viral diseases. However, further research is needed to fully understand the mechanisms by which MIF influences viral pathogenesis and the potential benefits and risks of targeting MIF in the context of viral infections. In addition to its potential therapeutic applications, MIF could also play a role in the diagnostic and prognostic aspects of the treatment of viral diseases [84,85].

## 4. Fungal Infections

MIF has also been shown to be involved in fungal infections. In their study, Stojanovic et al. demonstrated the role of MIF in the infection with *Aspergillus fumigatus* [86]. MIF-deficient mice showed a comparatively higher mortality when infected. Neutralization of MIF in WT mice with a specific inhibitor also negatively affected survival. While IFN-γ and IL-17 increased in WT mice during infection, they remained unchanged in MIF-deficient mice post-infection. An opposite tendency was observed in the secretion of IL-4, which was increased in MIF-deficient mice after infection compared to WT mice.

In their study, Mirkov et al. demonstrated that MIF acts as a resistance factor in sublethal infection with *Aspergillus fumigatus* and promotes the clearance of fungi in visceral organs and the brain [87].

Nicolo et al. demonstrated in their research that in mice infected with *Candida albicans*, the pro-inflammatory immune response was lower after an injection of anti-MIF IgG than after the administration of control IgG [88]. In addition, the mortality rate was increased in mice receiving anti-MIF IgG.

Xu et al. addressed the role of MIF in fungal keratitis in their study [89]. They showed that MIF as well as TNF-α and IL-6 are increased by *Aspergillus fumigatus*. Using a specific inhibitor of MIF, TNF-α and IL-6 were reduced, as was the proinflammatory response. MIF deficiency was found to be a protective factor in the study.

Overall, while MIF has been shown to play a complex role in fungal infections, the exact mechanism of action at work and the extent of the MIF contribution to the infection remain under investigation. Overall, additional research is needed to fully understand its role in fungal infections.

## 5. Parasitic Infections

In a study, Flores et al. investigated the role of MIF in the immune response against *Toxoplasma gondii* [39]. The authors demonstrated that MIF played a protective role. Conversely, MIF-deficient mice produced fewer proinflammatory cytokines, had more severe organ damage, and succumbed more rapidly to infection than WT mice.

An opposite trend was observed in a study on *Plasmodium yoelii* infections [90]. In this research, MIF-deficient mice showed lower parasitemia and delayed host mortality. Seven days post-*Plasmodium yoelii* 17XL infection, higher levels of IL-4, IL-10, IL-12, and IL-17 were observed in MIF-deficient mice than WT mice. In contrast, IFN-γ production was higher in WT mice.

A similar tendency was seen in the immune response to *Nippostrongylus brasiliensis* [91]. Here, MIF-deficient mice exhibited a lower parasite load as well as lower IL-6 induction seven days after infection than WT specimens. The mesenteric lymph nodes of MIF-deficient mice also showed higher GATA3 expression seven days post-infection. GATA3 is a Th2 transcription factor that regulates the development, maintenance, and proliferation of T cells and controls innate lymphoid cells [92]. WT mice treated with an MIF tautomerase inhibitor showed enhanced parasite clearance [91].

Certain protozoan parasites such as Entamoeba, Toxoplasma, and Plasmodium secrete an MIF cytokine homolog as a virulence factor that leads to host damage and exacerbates the disease [93,94,95,96,97,98,99,100,101,102]. In their study, Chen et al. investigated the effect of MIF in *Trichomonas vaginalis* under nutrient stress. A homologous MIF protein of *Trichomonas vaginalis* (TvMIF) was shown to be a survival factor during nutrient stress, and the overexpression of MIF as well as addition of recombinant human macrophage migration inhibitory factor increased parasite survival [103]. Furthermore, serum starvation was shown to increase the secretion of TvMIF, and the presence of TvMIF in serum starvation inhibited apoptosis through ROS suppression. Moreover, the gene knockout of TvMIF was shown to negatively affect parasite survival.

Liu et al. demonstrated the protective effect of the recombinant *Toxoplasma gondii* MIF protein vaccine (rTgMIF) after infection with *Toxoplasma gondii* in mice [104]. Immunization resulted in an increase in IFN-γ levels and a slight increase in IL-4 levels, indicating a shift toward a Th1-type immune response. Mice immunized with rTgMIF had significantly prolonged survival compared to controls in cases of acute infection with *Toxoplasma gondii* and significantly fewer brain cysts in instances of chronic infection with *Toxoplasma gondii*. rTgMIF could therefore be a candidate for vaccination and protection against *Toxoplasma gondii* [104].

**Figure 3:**
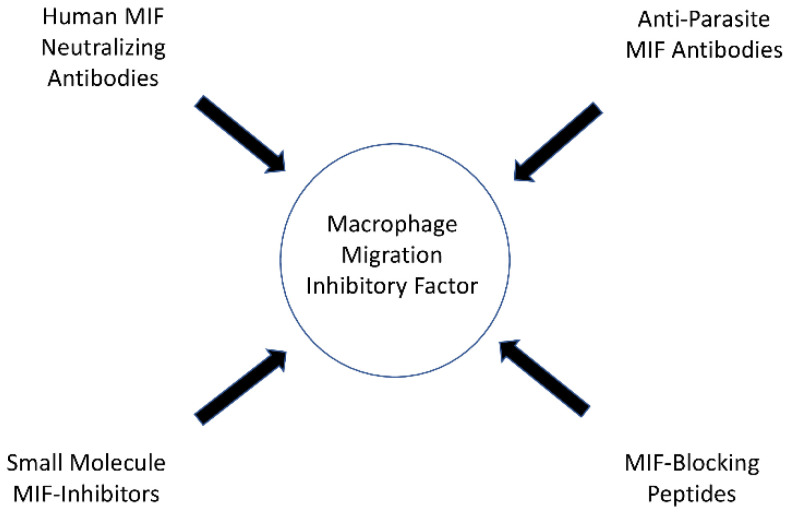
Possible options of MIF targeting drugs.

In their study, Ghosh et al. demonstrated that mice treated with metronidazole and anti-*Entamoeba histolytica* MIF antibodies exhibited reduced tissue damage and intestinal inflammation when infected with *Entamoeba histolytica* than mice treated with metronidazole alone [93]. The anti-parasite MIF-blocking antibodies did not cross-react with human MIF in the study, and therefore specific anti-parasite MIF-blocking antibodies may offer therapeutic benefits in the future, although additional research is needed [93]. An overview of the possible options of MIF-targeting drugs is presented in Figure 3.

## 6. Sepsis

The pathogenesis of sepsis is complex and involves the dysregulation of the immune response, leading to widespread inflammation and tissue damage. Bacterial lipopolysaccharide (LPS) is a component of the outer membrane in Gram-negative bacteria and is the most potent microbial mediator in the pathogenesis of sepsis [105,106]. In 1993, Bernhagen et al. showed that MIF is released by anterior pituitary cells in response to LPS, thereby indicating the possibility of MIF serving as a biomarker in sepsis [15,107]. Cecal ligation and puncture (CLP) is the most commonly used model to study sepsis [108]. *Escherichia coli* represents the most common Gram-negative pathogen of sepsis [16,109]. Using two models in mice, one triggered after an intraperitoneal injection of *Escherichia coli* and the other after CLP, Calandra et al. in 2000 reported an increase in MIF. This was found to occur first in the peritoneal cavity, and subsequently in the systemic circulation [16]. A protective effect of anti-MIF antibodies was also found in both models. However, the injection of exogenous MIF with *Escherichia coli* increased mortality compared to the injection of *Escherichia coli* alone.

From this point, several studies have been conducted on the role of MIF in sepsis [61,110,111,112,113,114,115,116]. In their study, Tilstam et al. found an increase in MIF levels after CLP [61]. MIF-deficient mice were also found to have delayed lethality and a threefold higher survival compared to WT mice. MIF-deficient mice exhibited reduced hypothermia, lower plasma creatine kinase levels, and lower plasma levels of several inflammatory cytokines (TNF- α, IL1-α, and IL1-β) after CLP treatment, while IL-10 levels were increased in the cases of peritoneal lavage. Furthermore, the study showed an association between a reduced number of small peritoneal macrophages (SPM) and survival after CLP. The number of SPM was in turn decreased in MIF-deficient mice, and the transference of SPM to MIF-deficient mice had a negative effect on survival [61].

A 2021 meta-analysis by Toldi et al. showed that blood MIF levels in patients with sepsis are significantly higher than those in patients with non-septic systemic inflammation or healthy control patients [117]. In addition, MIF has been shown to have prognostic value, as blood MIF levels are higher in nonsurviving sepsis patients than in surviving patients and in severer forms of sepsis compared with less severe forms of sepsis [117].

In their study, Tohyama et al. showed that protection and thus improved survival in mice against sepsis can be achieved by the use of autoantibodies against MIF induced by a DNA vaccination [118]. The research of Al-Abed et al. showed that an inhibitor of MIF tautomerase improved survival in sepsis in mice [119]. Therefore, it can be concluded that MIF has emerged as a potential biomarker and therapeutic target in sepsis. Research has shown that MIF levels are significantly elevated in patients with sepsis and that higher levels are associated with worse clinical outcomes. Further research is needed to fully understand the role of MIF in sepsis and to develop effective treatments for this life-threatening condition.

## 7. D-Dopachrome Tautomerase

Besides MIF, D-dopachrome tautomerase also represents a cytokine that plays a key role in the host immune response [120]. D-DT gene is also located on chromosome 22q11.2. D-DT is a homolog of MIF, has a relatively similar overall structure and is also called MIF-2 [17,120,121]. Compared to MIF, D-DT expression was demonstrated in fewer localizations, which included cardiomyocytes, hepatocytes, and dendritic cells as well as the bronchial and intestinal epithelium [66]. D-DT differs structurally from MIF by lacking the pseudo (E)LR domain essential for MIF’s chemokine function and the CXXC redox motif found in MIF [122]. While there is limited research on the regulation of D-DT transcription, one study has shown that its upregulation under hypoxia is dependent on a specific hypoxia-responsive element [66,123]. The amino acid similarity compared to MIF is limited, and D-DT is not as well understood in terms of its function as its more famous family member [17]. In addition to CD74, D-DT can also interact with CXCR7 [17]. MIF and D-DT do have some common catalytic and immunological functions—they trigger pro-inflammatory cascades via ERK-1/2-MAP kinases, and they counteract the anti-inflammatory effect of glucocorticoids [122]. There are also additive effects between MIF and D-DT, such as in the recruitment of neutrophils to the lungs [124]. In contrast, MIF and DDT play a different role in physiological processes such as adipogenesis and correlate differently with obesity [122]. D-DT has so far shown interesting results when applied in studies on spinal cord injury, amyotrophic lateral sclerosis, multiple sclerosis, tumors, skin diseases, heart failure, myocardial infarction, alveolar repair, orthotopic liver transplantation, and sepsis [61,121,125,126,127,128,129,130,131,132,133,134].

Merk et al. conducted a study and demonstrated that exposure to *Escherichia coli* LPS led to an increase in D-DT levels in both cultured macrophages and mice [40]. Peak levels were observed after 16 h in cultured macrophages, while the peak occurred 24 h after stimulation in mice. In addition, the effect of anti-D-DT antibodies on LPS administration in mice was investigated. The authors found that immune neutralization of D-DT strongly reduced mortality. While the concentration of IL-10 increased in the antibody-treated group, the levels of TNF-α, IFN-γ, and IL-1β decreased in this group compared with the control group. Merk et al. also demonstrated that patients with sepsis have higher serum D-DT concentrations than healthy control patients. In addition, D-DT also provides a prognostic value with respect to disease severity.

In 2021, Tilstam et al. confirmed an increase in D-DT levels in mice after CLP-induced sepsis [61]. In both peritoneal lavage fluid and systemic circulation, D-DT levels increased after CLP compared with cases of sham operation. In contrast to the survival benefits achieved after the immune neutralization of D-DT in the study by Merk et al., D-DT deficiency was not found to be a protective factor after the application of CLP in the study by Tilstam et al. [40,61]. This phenomenon was examined in this study via the use of mice lacking the D-DT gene [61].

In meta-analyses, obesity has been shown to be associated with better clinical outcomes in sepsis [135,136]. Kim et al. investigated the role of MIF and MIF-2 (D-DT) in white adipose tissue using an endotoxemia model [137]. They also confirmed an increase in D-DT in intraperitoneal fluid after LPS injection in mice. In visceral white adipose tissue, D-DT protein and D-DT mRNA levels decreased after LPS injection, whereas MIF protein and MIF mRNA expression increased after injection. While no difference was observed in the cells of the stromal vascular fraction between the endotoxemia and control groups with respect to D-DT levels, the adipocytes had significantly lower D-DT levels in this study. Furthermore, Kim et al. showed that knockout of the D-DT gene in adipose tissue macrophages resulted in a pro-inflammatory phenotype in adipose tissue macrophages, whereas MIF deficiency resulted in an anti-inflammatory phenotype. The relationship between MIF and D-DT/MIF-2 in adipocytes could therefore be inverted in sepsis.

Overall, although D-DT has been shown to play a role in the host immune response and has been associated with various diseases, including sepsis, its exact function and role in the immune response to individual microorganisms are yet to be fully understood. Compared to MIF, less is known about the functioning of D-DT, and further research is needed to elucidate its role in the immune system and explore its potential as a therapeutic target in various infections.

## 8. Conclusions

MIF has emerged as a fascinating and versatile cytokine with a controversial role in various types of infections and sepsis. As outlined in this review, MIF can have a dual function, acting as both a protective factor in host immune responses and, in some cases, exacerbating host injury. This duality highlights the complex interplay between MIF and the intricate immune mechanisms involved in infectious diseases and underscores the need for personalized approaches to therapeutic interventions targeting MIF-related pathways.

Several promising strategies have been identified that target MIF as a therapeutic agent, including human MIF neutralizing antibodies, anti-parasite MIF antibodies, small molecule MIF inhibitors, or MIF-blocking peptides. Additionally, routes to administer MIF or MIF activity-augmenting small molecules have been discussed. These therapeutic options hold great potential for use in the treatment of several infectious diseases and other MIF-associated pathologies and need to be further explored and developed. In addition to therapeutic applications, MIF is also emerging as a promising diagnostic and prognostic tool for use in various infectious diseases. The use of MIF as a potential biomarker could revolutionize disease detection and monitoring in these infectious diseases.

The deeper we delve into the fascinating world of MIF and D-DT and their interaction with infectious diseases, the clearer it becomes that further research is needed to fully unravel the intricacies of these model cytokines. By exploring the nuanced role of MIF in infection, immunity, and disease progression, we can pave the way for a more refined and personalized approach to MIF-targeting drugs and their application in the diagnosis and treatment in infections.

## Figures and Tables

**Table 1:** Important studies of the MIF cytokine superfamily in infection and inflammation.

Authors	Year of Publication	Title	Type	Topic
Bernhagen et al. [15]	1993	MIF is a pituitary-derived cytokine that potentiates lethal endotoxemia	In vitro and in vivo	MIF in sepsis
Bozza et al. [37]	1999	Targeted disruption of migration inhibitory factor gene reveals its critical role in sepsis	In vivo	MIF in sepsis
Calandra et al. [16]	2000	Protection from septic shock by neutralization of macrophage migration inhibitory factor	In vitro and in vivo	MIF in sepsis
Mitchell et al. [38]	2002	Macrophage migration inhibitory factor (MIF) sustains macrophage proinflammatory function by inhibiting p53: regulatory role in the innate immune response	In vitro and in vivo	MIF in apoptosis
Bernhagen et al. [18]	2007	MIF is a noncognate ligand of CXC chemokine receptors in inflammatory and atherogenic cell recruitment	In vitro and in vivo	MIF in inflammation and atherosclerosis
Flores et al. [39]	2008	Macrophage migration inhibitory factor (MIF) is critical for the host resistance against *Toxoplasma gondii*	In vitro and in vivo	MIF in *Toxoplasma gondii*
Merk et al. [40]	2011	The D-dopachrome tautomerase (DDT) gene product is a cytokine and functional homolog of macrophage migration inhibitory factor	In vitro and in vivo	D-DT as a homolog of MIF
Das et al. [41]	2013	Macrophage migration inhibitory factor (MIF) is a critical mediator of the innate immune response to *Mycobacterium tuberculosis*	In vitro and in vivo	MIF in *Mycobacterium Tuberculosis*
Kim et al. [42]	2020	D-dopachrome tautomerase in adipose tissue inflammation and wound repair	In vitro and in vivo	MIF and D-DT in adipose tissue during endotoxemia

**Table 2:** Simplified overview of the effect of MIF in different bacterial infections.

Microorganism	Gram Stain	Author(s)	MIF Effect
*Mycobacterium* *tuberculosis*	Positive	Oddo et al. [67], Das et al. [41],	Protective
*Streptococcus pneumoniae*	Positive	Savva et al. [71], Kloek et al. [72]	Detrimental
*Clostridium difficile*	Positive	Jose et al. [73]	Detrimental
*Salmonella typhimurium*	Negative	Koebernick et al. [59]	Protective
*Pseudomonas aeruginosa*	Negative	Adamali et al. [74], Doroudian et al. [75]	Detrimental

**Table 3:** MIF effects in different viral infections.

Viral Pathogen	Author(s)	MIF Effect
Dengue Virus	Assunção-Miranda et al. [1] Chen et al. [78], Lai et al. [79]	Deleterious (Facilitation of replication, vascular leakage, immune modulation)
Human immunodeficiency virus type I (HIV)	Trifone et al. [62,65]	Deleterious (Cytokine induction, CD4^+^ T cell predisposition, increased Th17-like cell profile)
Influenza A Virus (IAV)	Smith et al. [81]	Deleterious (Increased viral load, inflammation, and mortality)
Respiratory Syncytial Virus (RSV)	de Souza et al. [82]	Protective (Increased viral clearance)

## Data Availability

No new data were created or analyzed in this study. Data sharing is not applicable to this article.
